# Sudden cardiac death in newly diagnosed non-ischaemic or ischaemic cardiomyopathy assessed with a wearable cardioverter-defibrillator: the German nationwide SCD-PROTECT study

**DOI:** 10.1093/eurheartj/ehaf668

**Published:** 2025-08-29

**Authors:** David Duncker, Eloi Marijon, Marco Metra, Olivier Piot, Marat Fudim, Uwe Siebert, Norbert Frey, Lars Siegfried Maier, Johann Bauersachs

**Affiliations:** Hannover Heart Rhythm Center, Department of Cardiology and Angiology, Hannover Medical School, Carl-Neuberg-Str. 1, 30625 Hannover, Germany; Division of Cardiology, European Georges Pompidou Hospital, Paris, France; Université Paris Cité, PARCC, INSERM U970, Paris, France; Cardiology, ASST Spedali Civili, Department of Medical and Surgical Specialties, Radiological Sciences, and Public Health, University of Brescia, Brescia, Italy; Department of Cardiology 2, Centre Cardiologique du Nord, Saint-Denis, France; Cardiology, Duke University, Durham, NC, USA; Duke Clinical Research Institute, Durham, NC, USA; UMIT TIROL—University for Health Sciences and Technology, Department of Public Health, Health Services Research and Health Technology Assessment, Hall in Tirol, Austria; Center for Health Decision Science, Departments of Epidemiology and Health Policy & Management, Harvard T.H. Chan School of Public Health, Boston, MA, USA; Program on Cardiovascular Research, Institute for Technology Assessment and Department of Radiology, Massachusetts General Hospital, Harvard Medical School, Boston, MA, USA; Department of Cardiology, Angiology, Pneumology, University Hospital Heidelberg, Heidelberg, Germany; Internal Medicine II, University Hospital Regensburg, Regensburg, Germany; Hannover Heart Rhythm Center, Department of Cardiology and Angiology, Hannover Medical School, Carl-Neuberg-Str. 1, 30625 Hannover, Germany

**Keywords:** Sudden cardiac arrest, NICM, Myocardial infarction, Coronary artery disease, Incidence, Dilated cardiomyopathy

## Abstract

**Background and Aims:**

Patients with newly diagnosed non-ischaemic cardiomyopathy (NICM) or myocardial infarction/coronary artery disease (MI/CAD) face an increased risk of sudden cardiac death (SCD) during the early phase of guideline-recommended medical therapy initiation and up-titration. Aim is to evaluate the risk in this population by assessing sudden cardiac arrest (SCA) due to ventricular tachycardia/ventricular fibrillation (VT/VF).

**Methods:**

All patients in Germany who received a wearable cardioverter-defibrillator (WCD) between December 2021 and May 2023 were enrolled in the observational multicentre SCD-PROTECT study (NCT06883383). The primary outcome was the incidence of SCA due to sustained VT/VF, measured by appropriate WCD-delivered treatments, and reported as events per 100 patient-years with 95% confidence intervals (CI). Secondary outcomes included inappropriate WCD treatments, all-cause mortality, adverse events, adherence to WCD use, and heart failure medication patterns.

**Results:**

In this cohort of 19 598 patients, the mean age (±standard deviation) was 58.6 ± 13.7 years for those with NICM and 64.2 ± 10.6 years for patients with MI/CAD. Female patients accounted for 23.8% of the NICM and 16.3% of the MI/CAD group. Left ventricular ejection fraction (LVEF) at study start was 26.9 ± 10.3% for NICM and 28.4 ± 8.0% for MI/CAD patients. The incidence rate of first appropriate treatment by WCD in NICM patients and MI/CAD patients was 6.10 (95% CI 5.31–7.00) and 8.64 (95% CI 7.41–10.05) events per 100 patient-years, respectively. Overall incidence density for all appropriate treatments was 8.53 (95% CI 7.36–9.88) and 14.98 (95% CI 12.69–17.65) per 100 patient-years in the respective groups. Improvement in LVEF to >35% was observed in 53.5% of NICM patients and 51.7% of MI/CAD patients over a mean of 65.9 ± 43.8 days. 36.2% of patients were implanted with a cardioverter-defibrillator at the end of WCD use. Total mortality was 0.8%. Inappropriate shocks occurred in 0.5% of patients.

**Conclusions:**

The SCD-PROTECT study highlights a substantial risk of SCA due to VT/VF during the early phase of guideline-recommended medical therapy optimization in patients with newly diagnosed reduced LVEF, regardless of ischaemic or non-ischaemic origin. The WCD provided SCD protection, the LVEF could improve to >35% in the majority of these patients and can therefore serve as risk stratification across both aetiologies.

## Structured Graphical Abstract

**Structured Graphical abstract:**
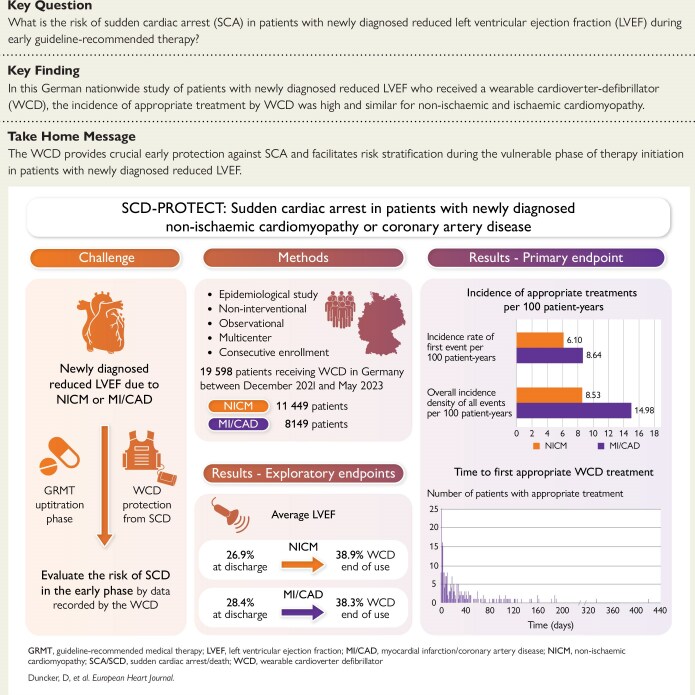

## Introduction

Sudden cardiac death (SCD) remains a leading mode of mortality worldwide, with an annual incidence estimated between 50 and 100 per 100 000 inhabitants in Europe, Australia, and North America.^1^ In Germany, the annual incidence of SCD is estimated at 81 per 100 000 persons, with a higher incidence in men (69%) compared to women (31%).^2^

The risk for SCD is notably increased in patients with reduced left ventricular ejection fraction (LVEF) to <30–40% due to either (i) myocardial infarction (MI) or coronary artery disease (CAD)^3,4^ or (ii) newly diagnosed non-ischaemic cardiomyopathy (NICM).^5^ Current guideline-recommended medical therapy (GRMT) for at least 3 months is mandatory for patients with a newly diagnosed LVEF ≤35%. If LVEF remains ≤35% or lower after this period and therefore the risk for SCD persists, an implantable cardioverter-defibrillator (ICD) is recommended (Class IIa recommendation) for primary prevention of SCD.^5–7^ During the early phase of GRMT up-titration, a wearable cardioverter-defibrillator (WCD) can serve as a temporary protective option, offering continuous arrhythmia monitoring and treatments for life-threatening ventricular arrhythmias.^8,9^ However, robust data on SCD risk during this vulnerable period, particularly in newly diagnosed NICM or early post-MI settings, remain limited.

The SCD-PROTECT study was designed to address this gap by providing real-world nationwide observational evidence on the risk of sudden cardiac arrest (SCA) in contemporary patients with NICM and MI/CAD during the initial months of medication optimization, and to better define the role of WCD use in this clinical window.

## Methods

### Patient selection

The SCD-PROTECT was a non-interventional, observational, multicentre epidemiological study that included all consecutive patients who were prescribed a WCD (LifeVest®, ZOLL, Pittsburgh, PA, USA) across 946 institutions in Germany. Patient enrolment began retrospectively in December 2021. From January 2023 to May 2023, participants were enrolled prospectively under the same study protocol. This combined retro-/prospective study design ensured achievement of the targeted sample size while capturing the most up-to-date data on SCA. The observation period in each patient (time zero) started with the first electrocardiogram (ECG) recording of the WCD and terminated with end of WCD use of the individual patient. As this was an observational study with the intention to capture the real-world situation of routine practice, no study-specific follow-up visits or diagnostic or monitoring procedures were scheduled outside of routine practices. The WCD and all medical treatments were prescribed as part of the usual clinical practice of the participating centres.

Inclusion criteria were prescription of a WCD due to high or uncertain SCD risk, patient-specific customization of the WCD, availability of a baseline ECG recording, and written informed consent. No exclusion criteria were applied.

All participants provided written consent for the use of data obtained from the manufacturer database and clinical records (e.g. discharge summaries) for research purposes. The study was conducted in accordance with the Declaration of Helsinki and was approved by the local ethics committee at Hannover Medical School (1732-BO-S-2025). The trial was registered with ClinicalTrials.gov ID NCT06883383.

### Data sources

The data sources in this study were derived exclusively from the manufacturer database and the remote Patient Management System (ZPM, ZOLL). The manufacturer database systematically captures all patients in Germany prescribed a WCD and included supplemental clinical information such as hospital discharge letters and medical reports. These documents were used to extract demographic characteristics, treatment data, and clinical history (e.g. indication for WCD, LVEF, or prior hospital admissions). All-cause mortality during the WCD wear period was also captured and included in the analysis. The ZPM provided detailed WCD recordings, which were used to evaluate appropriate and inappropriate treatments, as well as compliance (daily wear time) for each patient during the observation period. Data extraction from the manufacturer database (medical history, medical reports, discharge letters, diagnostic examination results) into a study database was performed by the local research team. Validation of WCD indication was carried out independently by cardiologists not involved in data collection. Similarly, the classification of treatments as appropriate or inappropriate was based on predefined ECG criteria and adjudicated independently by at least two medical experts. The merging of therapeutic data with diagnostic classification and other study-relevant variables was performed by a separate, independent reviewer to maintain objectivity. The authors affirm that all eligible patients were consecutively included in the study with all their available data. The authors were given unrestricted access to the data.

All patients were exclusively categorized into either NICM or MI/CAD sub-aetiologies. NICM subtypes were defined according to previous literature and included: (i) dilated cardiomyopathy (DCM) total, with further differentiation in DCM without ischaemic involvement or DCM with probable ischaemic involvement; (ii) tachycardiomyopathy; (iii) toxic cardiomyopathy; (iv) myocarditis (total, acute or non-acute); (v) Takotsubo syndrome; (vi) sarcoidosis; and (vii) NICM of unknown origin. Furthermore, we distinguished (viii) genetic indication and (ix) others. The DCM subgroup without ischaemic involvement includes all DCM patients without coronary heart disease and no pathological findings at coronary angiography. In the DCM subgroup with probable ischaemic involvement patients with coronary heart disease detected in any prior or current coronary angiography or prior MI was permitted, however no percutaneous coronary intervention (PCI) or coronary artery bypass graft (CABG), aligned with the previously described definitions.^10^ The ‘others’ category combined smaller subgroups with fewer than 50 patients each, including haemochromatosis, amyloidosis, left ventricular hypertrabeculation (formerly described as left ventricular non-compaction), hypertrophic cardiomyopathy (HCM), long QT syndrome or patients with peripartum cardiomyopathy, here patients with LVEF of >35% could be included if the underlying aetiology *per se* defined them as a high-risk group. Myocardial infarction/coronary artery disease sub-aetiologies included MI without procedure, with PCI or with CABG, and CAD with PCI or CABG.

### Primary and secondary endpoints

The primary endpoint was the incidence of SCA due to sustained ventricular tachycardia or ventricular fibrillation (VT/VF), presented as (i) incidence rate of first appropriate treatment (corresponding to the number of patients with at least one appropriate treatment) per 100 patient-years at risk; (ii) the overall incidence density for all appropriate therapies per 100 patient-years at risk (corresponding to all appropriate treatments); and (iii) cumulative incidence as percentage of patients with at least one appropriate treatment during the study period (indicated by median).

Incidence rate of first appropriate therapy was calculated as the number of first shock events divided by total observation time at risk for a first event (i.e. patients were censored after first event), multiplied by 100. In contrast, overall incidence density for all appropriate therapies included all appropriate shock events, as some patients had more than one appropriate shock, and patients were not censored after the first event. Cumulative incidence was calculated as the number of patients with at least one appropriate therapy during the study period divided by the number of all patients included, multiplied by 100.

Secondary endpoints included the cumulative incidence of inappropriate treatments (%), all-cause mortality during the observation period (%), and the risk of adverse events (%).

Exploratory endpoints comprised the trajectory of LVEF during follow-up, the proportion of patients who subsequently received an ICD, WCD compliance (measured as daily wear time), total duration of WCD use, and time from WCD initiation to the first WCD treatment.

### Statistical analysis

Categorical variables are described as numbers and percentages, continuous variables with means with standard deviations, median and interquartile range (IQR), and time to event data as incidence densities per person time at risk. Uncertainty is expressed using 95% confidence intervals (CI). Standardized mean differences (SMD) were calculated instead of *P*-values due to the large sample sizes. Cohen's d values for quantitative variables and Cohen's h values for qualitative variables were calculated as SMD. Sample size calculation was based on the binomial distribution. For sample size calculation a true appropriate WCD treatment percentage of 1.2% was assumed, resulting in a sample size of 8928 patients per indication needed to observe a minimum observed proportion (MOP) of 1.0% (89 events) with a probability of at least 95% (95% CI 0.008–0.0123). The error probability, that is, the probability of obtaining less than the desired MOP was set at 5%. Since two separate groups (patients with NICM and MI/CAD) were to be included, a total of about 18 000 patients was required. The calculations were performed with SAS EG 9.4 (SAS Institute Inc., Cary, NC, USA).

## Results

**Figure 1:**
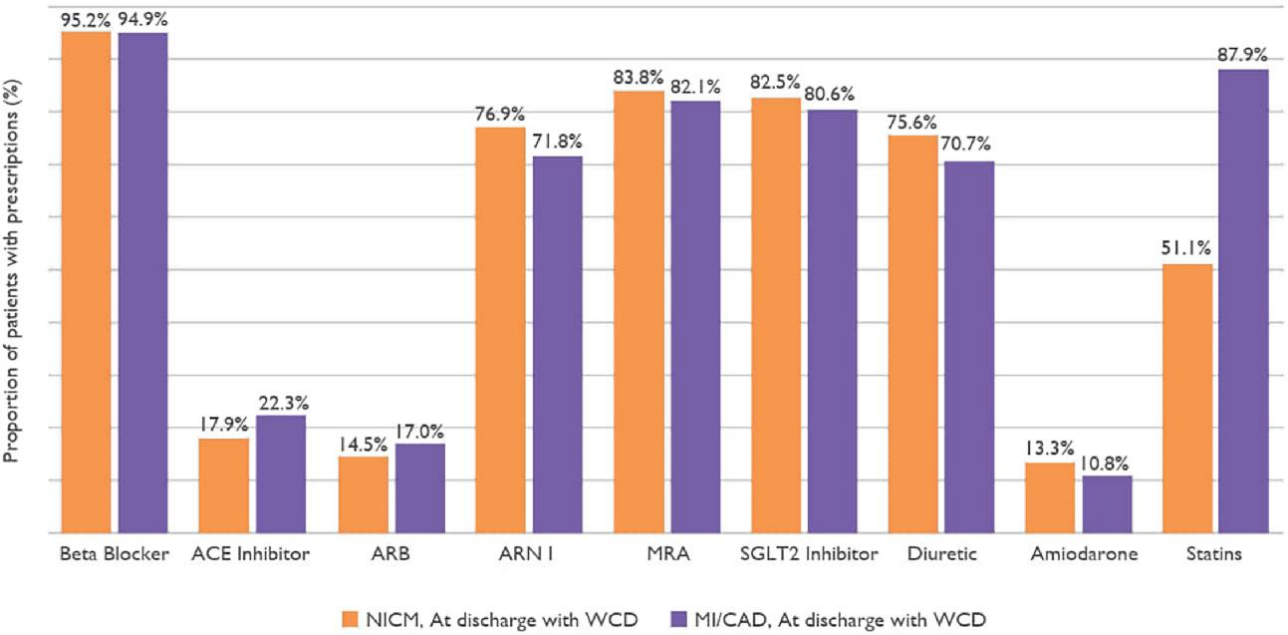
Medication at hospital discharge with wearable cardioverter-defibrillator. ACE, angiotensin converting enzyme; ARB, angiotensin receptor blocker; ARNI, angiotensin receptor–neprilysin inhibitor; CAD, coronary artery disease; MI, myocardial infarction; MRA, mineralocorticoid receptor antagonist; NICM, non-ischaemic cardiomyopathy; SGLT, sodium–glucose-co-transporter

A total of 19 598 patients were included in this consecutive cohort, comprising 11 449 patients with NICM and 8149 patients with MI/CAD. A total of 13 626 (69.5%) patients were retrospectively enrolled, while 5972 (30.5%) patients were prospectively enrolled. Baseline characteristics, cardiovascular history, and medication at the time of WCD prescription are summarized in *Table 1*. The mean age of the overall cohort was 60.9 ± 12.8 years, with patients with NICM being younger (58.6 ± 13.7 years) than those with MI/CAD (64.2 ± 10.6 years). Men represented 79.3% in the total population, 76.2% in NICM group, and 83.7% in the MI/CAD group. The median WCD wear duration was 62 days (IQR 33.0–90.0 days) for NICM and 61 days (IQR 35.0–89.0 days) for MI/CAD. Daily median WCD wear time was high across both groups: 23.4 h (IQR 21.4–23.8 h) for NICM and 23.5 h (IQR 22.1–23.8 h) for MI/CAD (*Table 2*). Medication at hospital discharge is presented in *Figure 1*.

**Table 1:** Baseline characteristics (*n* = 19 598), arrhythmias during hospital stay, and medication at discharge

	Total (*n* = 19 598)	NICM (*n* = 11 449)	MI/CAD (*n* = 8149)	SMD
Demographics				
Age, years, mean ± SD	60.9 ± 12.8	58.6 ± 13.7	64.2 ± 10.6	0.44
Female gender, *n* (%)	5350 (20.7)	2724 (23.8)	1329 (16.3)	0.19
LVEF at WCD fitting, %, mean ± SD	27.5 ± 9.4	26.9 ± 10.3	28.4 ± 8.0	0.16
Patients with arrhythmic events during hospital stay before WCD fitting^a^				
ns-VT, *n* (%)	1550 (12.4)	974 (13.4)	576 (11.1)	0.07
VT, *n* (%)	1200 (9.7)	690 (9.6)	510 (9.8)	0.01
VF, *n* (%)	555 (4.6)	175 (2.5)	380 (7.4)	0.23
Asystole, *n* (%)	160 (1.3)	96 (1.4)	64 (1.3)	0.01
Atrial fibrillation, *n* (%)	4797 (35.0)	3174 (39.1)	1623 (29.0)	0.21
Medical therapy at discharge, *n* (%)^a^				
Beta-blocker	14 274 (95.1)	8244 (95.2)	6030 (94.9)	0.01
ACE-inhibitor	2570 (19.7)	1332 (17.9)	1238 (22.3)	0.11
ARB	1414 (15.5)	759 (14.5)	655 (17.0)	0.07
ARNI	10 743 (74.7)	6410 (76.9)	4333 (71.8)	0.12
MRA	11 258 (83.1)	6611 (83.8)	4647 (82.1)	0.05
SGLT2 inhibitor	11 896 (81.7)	6925 (82.5)	4971 (80.6)	0.05
Diuretics	10 160 (73.5)	6067 (75.6)	4093 (70.7)	0.11
Amiodarone	1568 (12.3)	981 (13.3)	587 (10.8)	0.08
Statins	9578 (67.4)	4041 (51.1)	5537 (87.9)	0.84

ACE, angiotensin converting enzyme; ARB, angiotensin receptor blocker; ARNI, angiotensin receptor–neprilysin inhibitor; CAD, coronary artery disease; LVEF, left ventricular ejection fraction; MI, myocardial infarction; MRA, mineralocorticoid receptor antagonist; NICM, non-ischaemic cardiomyopathy; ns, non-sustained; SD, standard deviation; SGLT2, sodium–glucose-co-transporter 2; VF, ventricular fibrillation; VT, ventricular tachycardia; WCD, wearable cardioverter-defibrillator; SMD, standardized mean difference.

^a^Percentages refer to number of patients with reported data.

**Table 2:** Wearable cardioverter-defibrillator use and appropriate wearable cardioverter-defibrillator treatments

	Total (*n* = 19 598)	NICM (*n* = 11 449)	MI/CAD (*n* = 8149)	SMD
WCD wear time				
Number of days used, median (IQR)	62.0 (34.0–89.0)	62.0 (33.0–90.0)	61.0 (35.0–89.0)	
Number of days used, mean ± SD	65.9 ± 43.8	66.5 ± 44.8	64.9 ± 42.3	0.04
Number of days used, mean (censored at first appropriate shock)	65.7	66.4	64.8	
Wear time per day, h, median (IQR)	23.4 (21.7–23.8)	23.4 (21.4–23.8)	23.5 (22.1–23.8)	
Wear time per day, h, mean ± SD	21.5 ± 4.8	21.3 ± 4.9	21.8 ± 4.6	0.10
Appropriate WCD treatments				
Total number of appropriate WCD treatments	395	178	217	
Number of patients with appropriate treatment, *n* (%):				
At least one event	252 (1.3)	127 (1.1)	125 (1.5)	0.04
More than one event	72 (0.4)	34 (0.3)	38 (0.5)	0.03
Incidence of appropriate WCD treatments				
Number of patient-years^a^	3528	2083	1447	
Incidence rate of first appropriate treatment (treated patients) per 100 patient-years (95% CI low–high)	7.14 (6.43–7.93)	6.10 (5.31–7.00)	8.64 (7.41–10.05)	0.10
Number of patient-years	3538	2086	1449	
Overall incidence density of all appropriate treatments per 100 patient-years (95% CI low–high)	11.16 (10.09–12.34)	8.53 (7.36–9.88)	14.98 (12.69–17.65)	0.20

CAD, coronary artery disease; IQR, interquartile range; MI, myocardial infarction; NICM, non-ischaemic cardiomyopathy; WCD, wearable cardioverter-defibrillator; SMD, standardized mean difference.

^a^Wear time for incidence rate, censored after first treatment.

**Figure 2:**
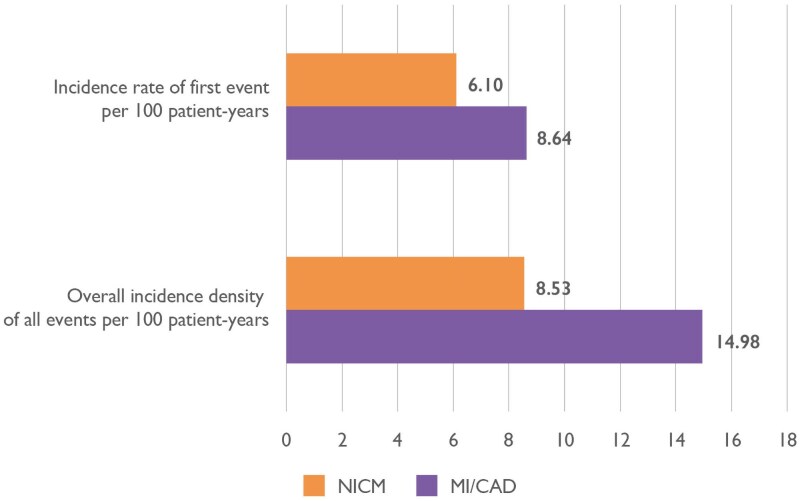
Incidence rate of first appropriate treatment and overall incidence density. MI/CAD, myocardial infarction/coronary artery disease; NICM, non-ischaemic cardiomyopathy

**Figure 3:**
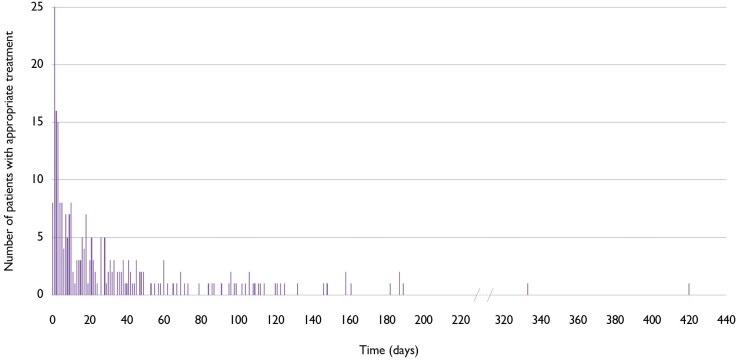
Time to first appropriate wearable cardioverter-defibrillator treatments

The incidence rate of first appropriate treatment in patients with NICM and MI/CAD was 6.10 (95% CI 5.31–7.00) and 8.64 (95% CI 7.41–10.05) events per 100 patient-years, respectively (*Figure 2*). The overall incidence density for all appropriate treatments in our study was 8.53 (95% CI 7.36–9.88) and 14.98 (95% CI 12.69–17.65) per 100 patient-years, respectively, with all but two events occurring within the first 200 days. The cumulative incidence of patients with at least one event was 1.3% over a median of 62 days (*Table 2*). *Figure 3* illustrates that the highest frequency of first appropriate shocks was observed in the initial days of WCD use, decreasing over time. In the NICM group, two events occurred late, at Days 333 and 420.

**Figure 4:**
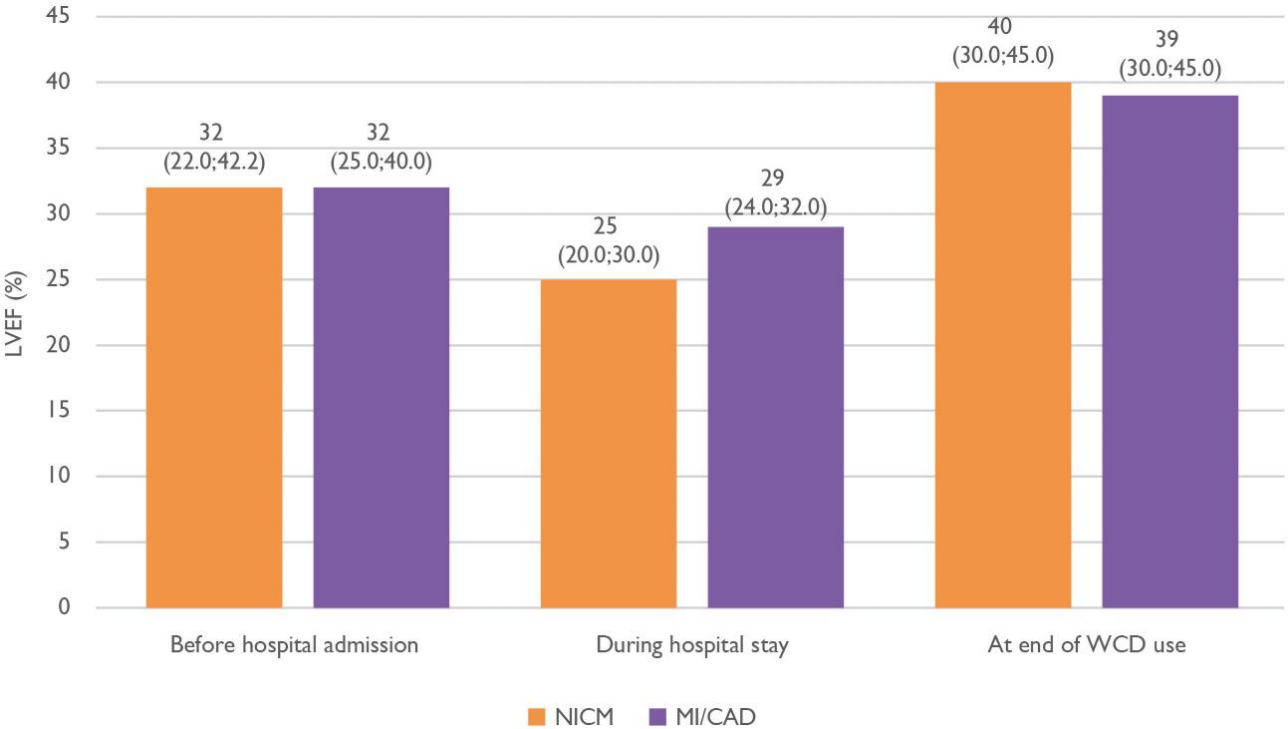
Median (interquartile range) left ventricular ejection fraction comparison in the main populations (non-ischaemic cardiomyopathy, myocardial infarction/coronary artery disease). IQR, interquartile range; LVEF, left ventricular ejection fraction; MI/CAD, myocardial infarction/coronary artery disease; NICM, non-ischaemic cardiomyopathy

Secondary endpoints are detailed in *Table 3*. Inappropriate treatments were rare, occurring in only 0.5% of patients. Mean LVEF at WCD prescription was 26.9 ± 10.3% in NICM and 28.4 ± 8.0% in MI/CAD (*Table 1*), improving during follow-up of 65.9 ± 43.8 days to above 35% in both groups (38.9 ± 10.8% and 38.3 ± 9.9%) as shown in *Figure 4*. Of 19 598 patients, there were 9861 patients with available ‘end of use reason’, which includes LVEF improvement, of which 5203 had documented LVEF improvement. Overall, mortality during WCD use was 0.8% (154 patients). Wearable cardioverter-defibrillator was discontinued due to LVEF improvement in 53.5% of NICM and 51.7% of MI/CAD patients. At the end of WCD use, ICDs had been implanted in 35.1% of patients with NICM and 37.8% with MI/CAD. Wearable cardioverter-defibrillator discontinuation due to skin irritation occurred in only 33 patients (0.3%).

**Table 3:** Secondary endpoints

	Total (*n* = 19 598)	NICM (*n* = 11 449)	MI/CAD (*n* = 8149)	SMD
Total number of inappropriate treatments	150	80	70	
Patients with inappropriate treatments, *n* (%):				
At least one inappropriate shock	105 (0.5)	55 (0.5)	50 (0.6)	0.01
More than one inappropriate shock	28 (0.1)	15 (0.1)	13 (0.2)	0.03
LVEF at end of WCD use, %, mean ± SD	38.7 ± 10.4	38.9 ± 10.8	38.3 ± 9.9	0.06
LVEF improvement >35%, *n* (%)^a^	5203 (52.8)	3079 (53.5)	2124 (51.7)	0.04
ICD implantation, *n* (%)^a^	3574 (36.2)	2020 (35.1)	1554 (37.8)	0.06
Death, *n* (%)	154 (0.8)	53 (0.5)	101 (1.2)	0.08

CAD, coronary artery disease; ICD, implantable cardioverter-defibrillator; LVEF, left ventricular ejection fraction; MI, myocardial infarction; NICM, non-ischaemic cardiomyopathy; SD, standard deviation; WCD, wearable cardioverter-defibrillator.

^a^Percentages refer to number of patients with reported data.

Detailed characteristics, LVEF evolution, and SCA due to VT/VF incidence by aetiological subgroups are provided in the supplement. Among all subgroups, the shortest median WCD wear time was observed in patients with Takotsubo syndrome (43 days, IQR 20.0–82.0), while the longest was seen in patients with MI and CABG (71 days, IQR 41.0–92.0). Median daily wear time across all groups exceeded 23 h, indicating high compliance. Implantable cardioverter-defibrillator implantation rates varied by NICM aetiology. An ICD was implanted in 39.8% of patients with DCM, 18.4% of patients with tachycardiomyopathy, 28.7% in patient with toxic cardiomyopathy, 25.2% in patients with myocarditis, 17.5% in patients with Takotsubo syndrome, 36.4% in patients with sarcoidosis, 32.0% in patients with NICM of unknown origin, 58.1% in patients with genetic indication, and 34.8% in patients with other aetiologies.

## Discussion

The SCD-PROTECT study provides robust, consecutive, and large-scale real-world observational data on the incidence of SCA due to VT/VF in patients treated with a WCD in Germany, encompassing over 19 500 patients and representing the largest WCD cohort published to date. The WCD delivers a treatment after about 1 min of sustained VT/VF on average. The response buttons, unique to the WCD, avoid treatments to conscious patients. Therefore, appropriate treatments by a WCD can be considered equivalent to the definition of a SCA caused by VT/VF. The study focused on patients with newly diagnosed low LVEF in NICM or MI/CAD, during the early phase of GRMT up-titration. The key finding is a substantial early-phase risk of SCA due to VT/VF, with an incidence rate for the first event of 6.10 (95% CI 5.31–7.00) and 8.64 (95% CI 7.41–10.05) per 100 patient-years in patients with NICM and MI/CAD, respectively (*Structured Graphical Abstract*).

These data challenge the commonly held assumption that patients with NICM are at a substantially lower—or even negligible short-term risk due to the higher likelihood of recovery, an assumption not supported by our findings. The adherence to the WCD was high, and different from that in the randomized controlled VEST trial.^11^ It seems that the randomized controlled trial protocol did not allow for monitoring, service, and personal interaction with patients and family, which, in contrast, is common in clinical practice in Germany.

A German registry study including 6043 WCD recipients (2010 to 2013) reported an incidence rate of 8.4 shocks per 100 patient-years across all indications.^12^ A meta-analysis covering WCD studies from 2008 to 2017 found a pooled incidence of 9.1 shocks per 100 patient-years.^13^ Wässnig *et al*. reported that among 2955 patients with NICM, 36 (1.2%) received appropriate WCD treatments.^12^ The WEARIT-France study, which included 1157 patients between 2014 and 2018 (82.1% patients with MI/CAD), reported an incidence of 7.2 shocks per 100 patient-years.^14^ A more recent meta-analysis showed incidence rates of 12, 16 and 19 per 1.000 patients for DCM, myocarditis, and Takotsubo syndrome, respectively.^15^

These results of previously published studies are roughly comparable with our results, indicating that the risk of SCD remained high even after introduction of novel medical treatments. Thus, our results do not support findings reported by Shen *et al*. that the risk for SCD substantially declined during the last 20 years due to new medical treatment options.^16^ As heart failure drugs need time for up-titration and for coming into effect, it is plausible that the effect of modern medication becomes visible in the later phase after some months, only—while there is no risk reduction in the first months. None of these studies reported time to first appropriate WCD treatment and thus no detailed data on the risk and length of the early vulnerable period for SCD during WCD use are available from published literature.

In our study, appropriate WCD treatments were concentrated in the early phase, with most events occurring within the first 30 days of WCD use, and virtually all between Day 0 to Day 200. Only two events occurred months later, both in the NICM group. This is a critical observation when assessing early risk and comparing study outcomes. In the DANISH study,^10^ 64 appropriate ICD shocks in 556 patients with NICM over an average follow-up of 67.6 months, resulting in a much lower incidence of 2.04 appropriate treatments per 100 patient-years. Our results suggest that SCA risk is markedly higher during the early phase of GRMT initiation than in the chronic, post-optimization phase—precisely the period covered in DANISH, where patients were already stabilized on optimal GRMT.^10^ Similarly, data from SCD-HeFT and Frodi *et al*. reported incidences of 5.1 and 1.5 appropriate treatments per 100 patient-years.^17,18^ These gaps highlight the importance of early risk identification and protection—something that earlier trials may not have captured.^8^

Within NICM, the DCM subgroup, representing the largest subgroup in our study, showed an incidence rate for the first event of 7.87 per 100 patient-years (cumulative incidence 1.5%). This aligns with earlier registry data (1.3% incidence, 9.7 events per 100 patient-years)^12^ and another study reporting cumulative incidences for appropriate shocks as high as 4%.^19^ In myocarditis patients, our observed incidence rate of 3.9 per 100 patient-years (cumulative incidence 0.8%) was slightly lower than prior findings (1.3%, or 6.6 per 100 patient-years).^12^ Among MI/CAD patients, the cumulative incidence of appropriate treatments was 1.9%, which is higher than the 1.3% observed in the VEST trial,^11^ which enrolled only within 7 days after hospital discharge. This time lag until WCD monitoring may have led to an underestimation of early SCA risk in VEST. Likewise, lower observed SCA incidences in MI/CAD patients with subsequent CABG may reflect the time lag, or the protective effect of complete revascularization. Data of a German registry with no overlap to our enrolment period showed a cumulative incidence of 2.2%, implying that our 500 patient’s cohort might be too small for statistically profound conclusions.^20^ Guideline-recommended medical therapy is key to improving LVEF and reducing mortality, although after a considerable time of drug optimization.^21^ Guideline-recommended medical therapy was prescribed at a high percentage in our study. Even the prescription of sodium–glucose co-transporter 2 inhibitors was high, implying a fast implementation of guideline recommendations by the German cardiologists. In the recent HF-OPT study, LVEF recovery was significantly associated with achievement of target doses of HF drugs by Day 90.^22^ In our cohort, LVEF improved from a median of 25% to 40% in NICM and from 29% to 39% in MI/CAD, consistent with prior reports of LVEF improvements in NICM, ranging from 34.8% to 40.8%.^23–26^ Improvement of LVEF to >35% after the initial 90 days of up-titration of GRMT is a key factor in the decision for primary prevention ICD implantation. As observed in the HF-OPT study, 46% of patients improved to LVEF >35% at Day 90% and 68% at Day 180.^22^ Notably, all appropriate WCD treatments in that study occurred within the first 90 days, further underscoring the early vulnerability phase.^22^ Despite efforts to enhance risk stratification beyond LVEF, including big data approaches,^27^ LVEF remains the only consistently validated independent predictor of SCA risk.^28^ In our cohort, 35.1% of NICM and 37.8% of MI/CAD patients received an ICD at the end of the WCD monitoring, mirroring findings from a systematic review (35%).^15^ The longer patients stay on GRMT, the more improve above an LVEF of 35%.^26^ The vulnerable phase of about 200 days found in our study suggests that 90 days of waiting, as currently often interpreted from guideline recommendations, might not be appropriate. The WCD seems to be suitable for protecting patients from SCD and in parallel providing data for risk stratification during that time to better inform the decision for primary preventive ICD indication.

The SCD-PROTECT revealed a vulnerable risk phase of about 200 days, after which almost no further SCA occurred. While we saw a substantial risk for SCD in the early phase, we cannot conclude to which extent the vulnerable phase of arrhythmic risk on one hand, and the underlying and possibly temporary risk of the specific aetiology, such as myocarditis, ischaemia, or heart failure on the other hand may have contributed to the risk. We also did not get information on the question of how the risk in patients with improved LVEF may develop in the long term. However, other studies suggest that the risk of such patients indeed stabilized.^19^

These findings reinforce the potential of WCDs not only as a protective tool, but also as a bridge towards more personalized, evidence-based ICD decision-making.^19,29^

Beyond its role as a life-saving device delivering appropriate shocks, the WCD should increasingly be recognized as a comprehensive cardiac monitoring tool capable of improving outcomes even without delivering therapy. Its continuous recording capabilities allow for the detection of specific heart rate patterns, variability, and early warning signs that may precede ventricular arrhythmias in the very short term. Using real-time physiological data provided by the WCD could help identify patients at imminent risk of life-threatening arrhythmias—within minutes or hours before an event—thereby enabling timely clinical intervention.^30,31^

### Limitations

Although SCD-PROTECT being the largest WCD cohort so far, we acknowledge limitations. Inclusion was limited to patients having been prescribed a WCD, which introduces a selection bias and may lead to underestimation of the overall SCA risk. By appropriate treatments we could only measure SCA due to VT/VF. There may have been other SCA from other causes. As the observation time ended with end of WCD use, we may have missed events occurring subsequently.

According to the study protocol, we did not exclude any patients with selected aetiologies, such as Takotsubo syndrome, myocarditis, and genetic channelopathies, which might introduce a bias in respect of the incidence of SCA in the NICM group. However, we found that by exclusion of those patients the incidence rate would only marginally rise.

Finally, part of the cohort was enrolled retrospectively; however, data collection followed protocols across both retrospective and prospective groups, minimizing potential bias. Data for some outcomes were not available for all patients. This may have led to bias in either direction. In contrast to previous studies,^32,33^ our dataset did not allow for a cost-effectiveness analysis due to limited data on healthcare resource utilization.

## Conclusion

The nationwide SCD-PROTECT study, comprising the largest WCD population to date, reveals a substantial early-phase risk of SCA in both, patients with newly diagnosed NICM and patients with newly diagnosed MI/CAD during GRMT up-titration. The incidence of appropriate WCD shocks was highest during the first weeks of use and exceeded rates reported in long-term ICD trials. Our study showed that the SCA risk was not substantially lower or negligible with non-ischaemic compared to ischaemic aetiologies. Importantly, a large proportion of patients with LVEF ≤35% at baseline improved in LVEF during WCD use, avoiding unnecessary ICD implantations. These findings underscore the critical need to protect high-risk patients during early therapy optimization.

## Supplementary Material

ehaf668_Supplementary_Data

## Declarations

### Data Availability

The data underlying this article cannot be shared publicly until all study results are published. Afterwards the data can be shared on reasonable request to the corresponding author.

### Pre-registered Clinical Trial Number

The pre-registered clinical trial number is ClinicalTrials.gov number: NCT 06883383.
