# Supplementary material for: Sudden cardiac death in newly diagnosed non-ischaemic or ischaemic cardiomyopathy assessed with a wearable cardioverter-defibrillator: the German nationwide SCD-PROTECT study
Source: Eur Heart J. 2025 Aug 29;46(43):4597–606. doi: 10.1093/eurheartj/ehaf668 (PMC12614993; doi:10.1093/eurheartj/ehaf668)
Supplement: ehaf668_Supplementary_Data [file ehaf668_supplementary_data.docx]

Supplementary tables to

Duncker et al.: Sudden cardiac death in newly diagnosed non-ischaemic or ischaemic cardiomyopathy assessed with a wearable cardioverter-defibrillator: the German nationwide SCD-PROTECT study

Supplementary Table 1: Sub-aetiologies – Patient characteristics and results of primary endpoint

|  | **n (%)** | **Age (years), mean ± SD** | **LVEF in % at WCD fitting, mean ± SD*** | **LVEF (%) at end of WCD use mean ± SD*** | **Treated patients with at least one event**  **n (%)** | **Incidence rate of first appropriate treatment (per 100 patient-years)** | **Overall incidence density of all appropriate treatments (per 100 patient-years)** |
| --- | --- | --- | --- | --- | --- | --- | --- |
| **NICM** | **11,449** |  |  |  |  |  |  |
| DCM | 6,462 (56.4) | 59.9±12.5 | 24.9±7.5 | 36.9±10.2 | 95 (1.5) | 7.88 | 11.35 |
| DCM without ischemic involvement | 3,937 (60.9) | 57.2±12.7 | 24.1±7.2 | 36.9±10.2 | 49 (1.2) | 6.51 | 8.48 |
| DCM with probable ischemic involvement | 2,525 (39.1) | 64.3±10.9 | 26.2±7.9 | 36.9±10.2 | 46 (1.8) | 10.18 | 16.14 |
| Tachymyopathy | 1,056 (9.2) | 63.3±11.0 | 26.6±8.6 | 42.7±9.5 | 13 (1.2) | 7.75 | 10.19 |
| Toxic cardiomyopathy | 351 (3.1) | 52.6±13.4 | 23.4±7.4 | 38.2±10.5 | 2 (0.6) | 3.19 | 3.20 |
| Myocarditis total | 888 (7.8) | 49.3±15.6 | 31.9±14.5 | 43.2±11.7 | 7 (0.8) | 3.89 | 4.45 |
| acute | 502 (56.5) | - |  |  | 5 (1.0) | 5.11 | 6.13 |
| non-acute | 386 (43.5) | - |  |  | 2 (0.6) | 2.44 | 2.44 |
| Takotsubo syndrome | 128 (1.1) | 61.3±13.6 | 32.3±12.0 | 46.2±12.1 | 0 | 0 | 0 |
| Sarcoidosis | 94 (0.8) | 52.3±12.3 | 35.1±13.7 | 43.3±9.8 | 2 (2.1) | 10.06 | 10.08 |
| NICM of unknown origin | 1,183 (10.3) | 60.2±12.8 | 27.2±9.9 | 40.0±10.2 | 1 (0.1) | 0.48 | 0.48 |
| Genetic indication | 144 (1.3) | 45.9±16.8 | 39.2±18.8 | 38.6±13.6 | 1 (0.7) | 3.54 | 7.08 |
| Others | 1,143 (9.9) | 55.5±16.1 | 33.8±14.5 | 42.8±11.6 | 6 (0.5) | 3.33 | 5.00 |
| **MI/CAD** | **8,149** |  |  |  |  |  |  |
| Acute MI | 4,860 (59.6) | 63.3±11.0 | 29.2±8.0 | 38.6±9.8 | 94 (1.9) | 10.91 | 19.56 |
| MI, without procedure | 511 (10.5) | - | - | - | 11 (2.2) | 11.83 | 29.04 |
| MI, with PCI | 3,790 (78.0) | - | - | - | 77 (2.0) | 11.69 | 18.47 |
| MI, with CABG | 559 (11.5) | - | - | - | 6 (1.1) | 5.43 | 18.04 |
| CAD with intervention | 3,289 (40.4) | 65.7±10.0 | 27.2±7.9 | 37.7±10.0 | 31 (0.9) | 5.30 | 8.21 |
| CAD with PCI | 2,335 (71.0) | - | - | - | 27 (1.2) | 6.60 | 10.50 |
| CAD with CABG | 954 (19.6) | - | - | - | 4 (0.4) | 2.28 | 2.85 |

CAD: Coronary artery disease; CABG: Coronary artery bypass graft; DCM: Dilated cardiomyopathy; ICD: Implantable cardioverter-defibrillator; LVEF: Left ventricular ejection fraction; MI: Myocardial infarction; NICM: Non-ischemic cardiomyopathy; PCI: Percutaneous coronary intervention; SD: Standard deviation; WCD: Wearable cardioverter-defibrillator

* Percentages refer to number of patients with reported data

Supplementary Table 2: Circumstances of death

| **Death (n=154)** | **n (%)** | **n (%) per subgroup** |
| --- | --- | --- |
| In hospital | 111 (72,1%) |  |
| Out of hospital | 43 (27,9%) |  |
| With WCD | 49 (31,8%) |  |
| Cardiac cause* |  | 21 (42,9%) |
| Non-cardiac cause |  | 12 (24,5%) |
| Unknown cause |  | 16 (32,7%) |
| Without WCD | 100 (64,9%) |  |
| Cardiac cause |  | 48 (48,0%) |
| Non-cardiac cause |  | 31 (31,0%) |
| Unknown cause |  | 21 (21,0%) |
| Unknown (all in hospital) | 5 (3,2%) |  |
| Cardiac cause |  | 0,0% |
| Non-cardiac cause |  | 40,0% |
| Unknown cause |  | 60,0% |

WCD: Wearable cardioverter-defibrillator

* No death due to VT/VF
